# New L-Serine Derivative Ligands as Cocatalysts for Diels-Alder Reaction

**DOI:** 10.1155/2013/217675

**Published:** 2013-12-05

**Authors:** Carlos A. D. Sousa, José E. Rodríguez-Borges, Cristina Freire

**Affiliations:** ^1^REQUIMTE, Departamento de Química e Bioquímica, Faculdade de Ciências da Universidade do Porto, Rua do Campo Alegre s/n, 4169-007 Porto, Portugal; ^2^Centro de Investigação em Química, Departamento de Química e Bioquímica, Faculdade de Ciências da Universidade do Porto, Rua do Campo Alegre s/n, 4169-007 Porto, Portugal

## Abstract

New L-serine derivative ligands were prepared and tested as cocatalyst in the Diels-Alder reactions between cyclopentadiene (CPD) and methyl acrylate, in the presence of several Lewis acids. The catalytic potential of the *in situ* formed complexes was evaluated based on the reaction yield. Bidentate serine ligands showed good ability to coordinate medium strength Lewis acids, thus boosting their catalytic activity. The synthesis of the L-serine ligands proved to be highly efficient and straightforward.

## 1. Introduction

The synthesis of bicyclic compounds has large significance due to their use as synthetic intermediates in the preparation of a vast variety of compounds of chemical, biological, and pharmaceutical interest [1, 2]. The most efficient and widely used method for the preparation of bicyclic compounds is the Diels-Alder reaction. Generally, activation by an electron-withdrawing group and a Lewis acid is required in order to achieve good conversion rates.

The acid catalyzed Diels-Alder reactions, namely, between cyclopentadiene (CPD) and acrylates, is well documented [1, 3–15], the most important used Lewis acids being Al(III), Fe(III) or boron complexes. The use of such strong acids is needed because ester dienophiles (as acrylate ones) are not very reactive [15, 16]. Depending on the reactions, the solvent used is also a factor to consider regarding both the Lewis acid solubility and the reaction media's polarity, dichloromethane being the most used solvent as combines both properties.

**Scheme 1:**
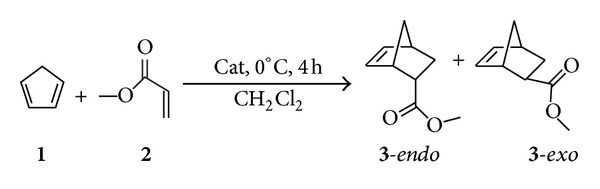
Lewis acid catalyzed Diels-Alder reaction between CPD (**1**) and methyl acrylate (**2**).

In this work, we studied the use of moderate strength Lewis acids as catalysts for Diels-Alder reaction between CPD (**1**) and methyl acrylate (**2**) in dichloromethane (Scheme 1), by complexing insoluble metal ions with novel L-serine derivative ligands, as alternative to the usual strong Lewis acids.

## 2. Results and Discussion

The work started with the study of several Lewis acids tested as catalysts in the Diels-Alder reaction between CPD (**1**) and methyl acrylate (**2**) using dichloromethane as solvent; the results are summarized in Table 1, as well as the reaction conditions.

As expected, in the absence of catalyst, the reaction did not take place (entry 1). For the catalyzed reactions, it is noteworthy the correlation observed between the Lewis acid strength and the yield of the reaction: the best results were achieved when the stronger Lewis acids AlCl_3_, FeCl_3_, and TiCl_4_ were employed (entries 2, 4 and 5, resp.). In comparison with AlCl_3_, a slight decrease in the reaction yield was observed when AlMe_3_ was used (entry 3). SnCl_4_ originated a lower yield than the previous mentioned strong Lewis acids (entry 6). For Cu(OTf)_2_ and ZnI_2_, which are moderate Lewis acids, the yields were quite low (entries 7, 8); nevertheless, their poor solubility in the solvent used (dichloromethane) may also explain these results. The increase of the reaction time (entries 9, 10) and of the amount of catalyst employed (entry 11) did not significantly change the results in terms of yield. In fact, similar results were observed in a previous work comprising the catalytic cycloaddition reaction between CPD and methyl glyoxylate oxime [17]. It is possible, however, that increasing the reaction time when the strong acids were used resulted in a decrease in the Diels-Alder reaction yields, probably due to decomposition of the final adduct (entry 12) [17, 18].

Considering this, we synthesized new L-serine derivative ligands as inexpensive ligands for application on the Diels-Alder reactions. Serine has two major advantages for this purpose: being cheap and readily available and having three functional groups that may be easily functionalized, thus allowing chemical and structural design. In fact, serine was earlier used for other purposes as evaluation as ligand for complex formation with Cd(II), complexation with Cu(II) for antibacterial studies, and used as recyclable catalyst for asymmetric aldol reactions [19–21]. In this work, L-serine derivative ligands were designed and used to coordinate the Lewis acids, in order to form *in situ* metal complexes that would be more soluble in dichloromethane than the original Lewis acids. In this context, only Cu(II) and Zn(II) salts (Cu(OTf)_2_ and ZnI_2_) were considered in the catalytic reaction. In fact, complexes of Zn(II) and Cu(II) are widely used as Lewis acids in Diels-Alder reactions [1].

**Scheme 2:**
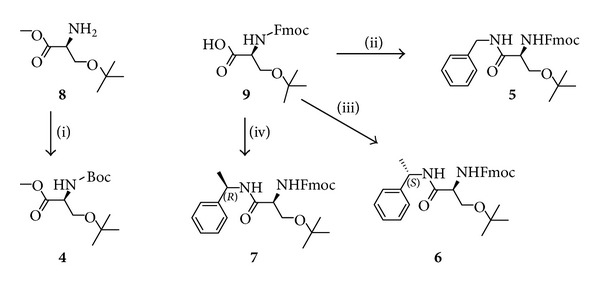
L-serine derivative ligands used in the Diels-Alder reaction between **1** and **2**. Reagents and conditions: (i) Boc_2_O, CH_2_Cl_2_, r.t., 2 h, 86%; (ii) benzylamine, DIPEA, TBTU, CH_2_Cl_2_, r.t., 2 h, 99%; (iii) (*S*)-1-phenylethylamine, DIPEA, TBTU, CH_2_Cl_2_, r.t., 2 h, 100%; (iv) (*R*)-1-phenylethylamine, DIPEA, TBTU, CH_2_Cl_2_, r.t., 2 h, 100%.

The chemical structures of the prepared L-serine derivative ligands **4**–**7** either from **8** or **9** (commercially available) are depicted in Scheme 2. Ligand **4** was effectively obtained by protecting the amine group of serine **8** with Boc; ligands **5**–**7** were prepared in excellent yields by reacting the respective amine with serine **9** in the presence of DIPEA and TBTU.

The Diels-Alder reaction represented in Scheme 1 was then tested in the presence of the ligands **4**–**7** and the results are summarized in Table 2. The reaction conditions were those represented in Scheme 1, except in the indicated cases.

It is noteworthy that ligand **8** completely deactivated the metal catalyst (entries 1, 2, and 3), with the strong catalytic effect of AlCl_3_ and FeCl_3_ (entries 2 and 4, Table 1) being totally suppressed. This suggests that the cation coordinates to the basic amine group of **6** and the resulting complex is not sufficiently acidic to catalyze the Diels-Alder reaction. The Boc-protection of serine **8** led to serine ligand **4**. The addition of **4** to a suspension of Cu(OTf)_2_ in dichloromethane did not result in a colored homogeneous solution, indicating that the complex **4**-Cu was not formed. Consequently, the reaction yield was drastically decreased (entry 4, Table 2) when compared with the use of Cu(OTf)_2_ alone (entry 9, Table 1), as the acid character of the Lewis catalyst was compensated with the basic character of amide **4**. The same reaction was performed with serine **9** and Cu(OTf)_2_. Similarly, the **9**-Cu complex was not formed, as indicated by the nonhomogenization of the solution. However, in this case the reaction yield was not significantly changed when compared with the use of Cu(OTf)_2_ alone (entry 5, Table 2, and entry 9, Table 1, resp.). This is in agreement with the acid character of **9**.

The bidentate serine derivative ligands **5**–**7** were also tested as cocatalysts. The complete solubilization and coloring subsequent to the addition of ligands **5**–**7** to a suspension of the Lewis acids [yellow for Zn(II) and blue for Cu(II)] point to a good metal to ligand complexation. By analyzing Table 2, it is possible to verify that the use of ligand **5** significantly improved the reaction yield (when compared with the use of Lewis acid alone). We further studied the influence of steric effects near to the ligand binding atom on the catalytic performance, by comparing the performance of **5** (ligand with a benzylamine moiety) with **6** [(*S*)-phenylethylamine moiety] and **7** [(*R*)-phenylethylamine moiety]. The results clearly showed that the methyl group of the phenylethyl moiety slightly blocks the approximation of the CPD to the dienophile-metal-ligand complex when it is at  *S*  configuration. On the other hand, the e.e. was slightly higher for **6** than for **7** (entries 9–12, Table 2). No significant effect was observed when it is at  *R*  configuration. The *endo*/*exo* ratio of **3** does not seem to significantly change with the used ligand.

Finally, the use of metal to ligand molar ratio of 1 : 2 led to similar results to the usual 1 : 1 molar ratio, suggesting that each metal ion coordinates to only one serine derivative ligand, contrary to what occurs with  *C*_2_  symmetry ligands [22]. In fact, BOX ligands proved to be quite ineffective in this reaction, leading to very low yields and low e.e. (see Supplementary data available online at http://dx.doi.org/10.1155/2013/217675).

## 3. Conclusions

L-serine based ligands showed good potential to be applied in Diels-Alder reactions, particularly if coordinated to moderate-strength Lewis acids such as Cu(OTf)_2_ and ZnI_2_. This is an alternative to the usage of strong Lewis acids such as AlCl_3_ or FeCl_3_, as serine derivative ligands proved to allow good yields at mild conditions are cheap and easy to prepare.

The results also anticipate that the change of the serine carboxylic residue by a chiral amide group may influence the stereochemistry of the Diels-Alder reaction, with this subject being currently under study and developed in our laboratory.

## 4. Materials and Methods

### 4.1. General Notes

All solvents were distilled and dried using standard methods. CPD was freshly bidistilled prior to use. All starting material and reagents were from commercial suppliers (Aldrich, Fluka, Bachem) and used without purification. Serine **8** was obtained by treatment of its commercial hydrochloride with triethylamine.

Flash column chromatography was performed on silica gel (60 Å, 230, 240 mesh) and analytical thin-layer chromatography (TLC) on precoated silica gel 60 F254 plates using iodine vapor and/or UV light (254 nm) for visualization. Melting points were determined on an electrothermal melting point apparatus and are uncorrected. Optical rotations were measured on a conventional thermostated polarimeter using a sodium lamp.

### 4.2. Experimental Procedure for Diels-Alder Reactions

To a suspension of catalyst (0.10 eq) in anhydrous dichloromethane (10 mL) at the conditions referred in Table 1, methyl acrylate (0.136 mL, 1.50 mmol) and CPD (0.125 mL, 1.51 mmol) were added. The mixture was left to react with stirring under argon atmosphere. After the reaction period, the mixture was treated by either the methods: (a) filtration through celite/silica with dichloromethane and (b) extraction from water with dichloromethane, followed by drying with anhydrous Na_2_SO_4_. The solvent was eliminated at reduced pressure. Whenever necessary, methanol was added to the dry crude in order to polymerize unreacted CPD. The formed polymer was triturated, filtered off, and washed with methanol. After evaporation of the volatiles, the crude was weighed and analyzed by NMR in order to evaluate both the reaction yield and the *endo*/*exo* ratio.

For Diels-Alder reaction catalyzed by a serine-metal complex to a suspension of catalyst (0.10 eq) in anhydrous dichloromethane (10 mL) at the conditions referred to as in Table 2, the ligand (0.10 eq) was added and the mixture was left to react under argon atmosphere into an ice bath during 30 min. After this period, methyl acrylate (0.136 mL, 1.50 mmol) and CPD (0.125 mL, 1.51 mmol) were added, with the subsequent procedure being similar to the previously described one.

(±)*-methyl bicyclo[2.2.1]hept-5-ene-2-endo-carboxylate *(**3*-*endo**).  ^1^H NMR (400 MHz, CDCl_3_): *δ* = 6.22 (dd, *J* = 5.7, 3.1 Hz, 1H, 5-H), 5.95 (dd, *J* = 5.7, 2.8 Hz, 1H, 6-H), 3.65 (s, 3H, OCH_3_), 3.20–3.24 (m, 1H, 1-H), 2.97 (dt, *J* = 9.3, 3.8 Hz, 1H, 2-H), 2.90–2.95 (m, 1H, 4-H), 1.93 (ddd, *J* = 12.0, 9.3, 3.7 Hz, 1H, 3-H), 1.41–1.48 (m, 1H, 3-H +  7_syn_-H), 1.27–1.23 (m, 1H,  7_anti_-H).

(±)*-methyl bicyclo[2.2.1]hept-5-ene-2-exo-carboxylate *(**3*-*exo**).^1^H NMR (400 MHz, CDCl_3_): *δ* = 6.16 (dd, *J* = 5.6, 2.9 Hz, 1H, 5-H), 6.13 (dd, *J* = 5.5, 3.0 Hz, 1H, 6-H), 3.72 (s, 3H, OCH_3_), 3.05–3.08 (m, 1H, 4-H), 2.23–2.28 (m, 1H), 1.53–1.57 (m, 1H) (other signals are superimposed).

### 4.3. Synthesis of L-Serine Derivative Ligands****

*(S)-methyl 3-(tert-butoxy)-2-((tert-butoxycarbonyl)amino) propanoate *(**4**).  A solution of **8** (0.200 g, 0.945 mmol) and Boc_2_O (0.207 g, 0.948 mmol) in anhydrous dichloromethane (5 mL) was stirred at room temperature under argon atmosphere overnight. Water was added (10 mL) and the organic phase was separated. The aqueous phase was extracted with dichloromethane (3 × 10 mL). The organic extracts were rinsed with brine, dried over anhydrous Na_2_SO_4_, and evaporated at reduced pressure, yielding a colorless oil that was purified by chromatographic column (eluent: Hex/AcOEt 1 : 1). Traces of Boc_2_O were eliminated by leaving the oil under high vacuum overnight. *η* = 86%.

^*1*^ *H NMR (400 MHz, CDCl*_*3*_). *δ* = 5.35 (d, *J* = 8.4 Hz, 1H, NH), 4.33–4.43 (m, 1H, CH), 3.79 (dd, *J* = 2.8, 8.9 Hz, 1H, OCH_a_CH_b_), 3.73 (s, 3H, OCH_3_), 3.56 (dd, *J* = 3.2, 9.0 Hz, 1H, OCH_a_CH_b_), 1.45 (s, 9H, C(CH_3_)_3_), 1.13 (s, 9H, C(CH_3_)_3_); ^13^C NMR (100 MHz, CDCl_3_): *δ* = 171.6 (COO), 155.7 (HNCOO), 79.9 (C(CH_3_)_3_), 73.4 (C(CH_3_)_3_), 62.2 (OCH_2_), 54.4 (CH), 52.3 (OCH_3_), 28.4 (C(CH_3_)_3_), 27.4 (C(CH_3_)_3_); ESI-MS: calculated for [C_13_H_25_NO_5_ + H]^+^ (M + H^+^) 276.17, obtained 276.22.

*Bidentated Serines *(**5**–**7**). To a solution of **9** (0.50 g, 1.3 mmol) in anhydrous dichloromethane (25 mL), TBTU (0.63 g, 2.0 mmol), DIPEA (0.34 mL, 2.0 mmol), and the corresponding amine (1.3 mmol) were added. The mixture was left to react at room temperature under argon atmosphere for 2 h. 10 mL of water was added and the organic phase was separated. The aqueous phase was extracted with dichloromethane (3 × 10 mL). The organic extracts were rinsed with brine, dried over anhydrous Na_2_SO_4_, and evaporated at reduced pressure, affording the expected serine derivative ligands **5**, **6**, or **7 **with 99% yield. No further purifications were needed.

*(S)-(9H-fluoren-9-yl)methyl (1-(benzylamino)-3-(tert-butoxy)-1-oxopropan-2-yl)carbamate *(**5**).  ^1^H NMR (400 MHz, CDCl_3_): *δ* = 7.79 (d, *J* = 7.5 Hz, 2H,  H_ar-Fluoren_), 7.62 (d, *J* = 7.4 Hz, 2H,  H_ar-Fluoren_), 7.39–7.45 (m, 2H_ar_), 7.28–7.38 (m,  7H_ar_), 6.91 (bs, 1H, NH), 5.79 (bs, 1H, NH), 4.47–4.57 (sl, 2H, CH_2_Ph), 4.44 (d, *J* = 6.8 Hz, 2H, COOCH_2_), 4.28 (sl, 1H, COCH), 4.24 (t, *J* = 7.0 Hz, 1H, CH_Fluoren_), 3.87 (dd, *J* = 8.6, 3.7 Hz, 1H, OCH_a_CH_b_), 3.42 (t, *J* = 8.0 Hz, 1H, OCH_a_CH_b_), 1.17 (s, 9 H, C(CH_3_)_3_); ^13^C NMR (100 MHz, CDCl_3_): *δ* = 170.2 (COO), 156.1 (HNCOO), 143.8 and 143.7 (2 × C_Fluoren_), 141.3 (2 × C_Fluoren_), 137.9  (C_ipso Ph_), 128.7 (2 ×  C_meta Ph_), 127.8 (2 ×  CH_Fluoren_), 127.6 (2 × C_ortho Ph_), 127.5  (C_para Ph_), 127.1 (2 ×  CH_Fluoren_), 125.0 and 125.1 (2 ×  CH_Fluoren_), 120.0 (2 ×  CH_Fluoren_), 74.2 (C(CH_3_)_3_), 67.0 (COOCH_2_), 61.9 (OCH_2_), 54.6 (CH), 47.2  (CH_Fluoren_), 43.6 (CH_2_Ph), 27.4 (C(CH_3_)_3_); ESI-MS: calculated for [C_29_H_32_N_2_O_4_ + H]^+^ (M + H^+^) 473.24, obtained 473.28; m.p. = 125–130°C;  [*α*]_D_^24°C^  = +25.0 (*c* 1, CHCl_3_).

*(9H-fluoren-9-yl)methyl ((S)-3-(tert-butoxy)-1-oxo-1-(((S)-1-phenylethyl)amino)propan-2-yl)carbamate *(**6**).  ^1^H NMR (400 MHz, CDCl_3_): *δ* = 7.78 (d, *J* = 7.5 Hz, 2H,  H_ar-Fluoren_), 7.61 (d, *J* = 7.4 Hz, 2H,  H_ar-Fluoren_), 7.29–7.45 (m,  9H_ar_), 7.03 (bs, 1H, NH), 5.83 (bs, 1H, NH), 5.17–5.07 (m, 1H, CHPh), 4.41 (d, *J* = 7.5 Hz, 2H, COOCH_2_), 4.20–4.28 (m, 2H, COCH +  CH_Fluoren_), 3.81 (dd, *J* = 8.5, 4.1 Hz, 1H, OCH_a_CH_b_), 3.32 (t, *J* = 8.2 Hz, 1H, OCH_a_CH_b_), 1.53 (d, *J* = 6.9 Hz, 3H, CH_3_), 1.15 (s, 9H, C(CH_3_)_3_); ^13^C NMR (100 MHz, CDCl_3_): *δ* = 169.2 (COO), 156.0 (HNCOO), 143.9  (C_ipso Ph_), 143.7 (2 ×  C_Fluoren_), 141.3 (2 ×  C_Fluoren_), 128.7 (2 ×  C_meta Ph_), 127.7 (2 ×  CH_Fluoren_), 127.4  (C_para Ph_), 127.1 (2 ×  CH_Fluoren_), 126.1 (2 × C_ortho Ph_), 125.1 (2 ×CH_Fluoren_), 120.0 (2 ×  CH_Fluoren_), 74.3 (C(CH_3_)_3_), 67.0 (COOCH_2_), 61.8 (OCH_2_), 54.0 (CH), 49.2 (CHCH_3_), 47.1  (CH_Fluoren_), 27.3 (C(CH_3_)_3_), 22.0 (CHCH_3_); calculated for [C_30_H_34_N_2_O_4_ + H]^+^ (M + H^+^) 487.25, obtained 487.26; wax;  [*α*]_D_^24°C^  = −2.9 (*c* 1, CHCl_3_).

*(9H-fluoren-9-yl)methyl ((S)-3-(tert-butoxy)-1-oxo-1-(((R)-1-phenylethyl)amino)propan-2-yl)carbamate *(**7**).  ^1^H NMR (400 MHz, CDCl_3_): *δ* = 7.78 (d, *J* = 7.5 Hz, 2H,  H_ar-Fluoren_), 7.61 (d, *J* = 7.5 Hz, 2H,  H_ar-Fluoren_), 7.28–7.45 (m, 9H_ar_), 6.96 (bs, 1 H, NH), 5.80 (bs, 1H, NH), 5.20–5.09 (m, 1H, CHPh), 4.43 (d, *J* = 6.9 Hz, 2H, COOCH_2_), 4.24 (m, 2H, COCH +  CH_Fluoren_), 3.86 (dd, *J* = 8.1, 3.4 Hz, 1H, OCH_a_CH_b_), 3.41 (t, *J* = 8.2 Hz, 1H, OCH_a_CH_b_), 1.52 (d, *J* = 6.9 Hz, 3H, CH_3_), 1.23 (s, 9H, C(CH_3_)_3_); ^13^C NMR (100 MHz, CDCl_3_): *δ* = 169.3 (COO), 156.0 (HNCOO), 143.9 and 143.7 (2 ×  C_Fluoren_), 143.0  (C_ipso Ph_), 141.3 (2 ×  C_Fluoren_), 128.7 (2 ×  C_meta Ph_), 127.7 (2 ×  CH_Fluoren_), 127.4  (C_para Ph_), 127.1 (2 ×  CH_Fluoren_), 126.0 (2 ×  C_ortho Ph_), 125.1 (2 ×  CH_Fluoren_), 120.0 (2 ×  CH_Fluoren_), 74.3 (C(CH_3_)_3_), 67.1 (COOCH_2_), 62.0 (OCH_2_), 54.4 (CH), 49.0 (CHCH_3_), 47.2  (CH_Fluoren_), 27.5 (C(CH_3_)_3_), 22.1 (CHCH_3_); calculated for [C_30_H_34_N_2_O_4_ + H]^+^ (M + H^+^) 487.25, obtained 487.26; m.p. = 147-148°C;  [*α*]_D_^24°C^  = +48.7 (*c* 1, CHCl_3_).

## Supplementary Material

This material contains NMR spectra and results of Diels-Alder reactions using BOX ligands.

## Figures and Tables

**Table 1:** Results of the Diels-alder reaction between **1** and **2**, yield, and *endo*/*exo* ratio of adduct **3**.

Entry	Catalyst	Reaction time/h	*η*/%	*Endo/Exo* ratio^c^
1	—	4	—	—
2	AlCl_3_		87	95/5
3	AlMe_3_		70	93/7
4	FeCl_3_		75	95/5
5	TiCl_4_		80	94/6
6	SnCl_4_		67	94/6
7	Cu(OTf)_2_		35	98/2
8	ZnI_2_		11	94/6
9	Cu(OTf)_2_^a^	20	38	85/15
10	ZnI_2_^a^		16	92/8
11	Cu(OTf)_2_^b^	2	32	94/6
12	AlCl_3_	20	53	91/9

The reactions were performed with 10% of catalyst, at 0°C in dichloromethane, except in the mentioned cases. ^a^0°C to room temp.; ^b^30 % of catalyst; ^c^measured by ^1^H-NMR.

**Table 2:** Results of the Diels-alder reaction between **1** and **2**, yield, *endo*/*exo* ratio of adduct **3**, and enantiomeric excess (e.e.).

Entry	Catalyst	Ligand	Reaction time/h	*η*/%	*Endo*/*exo* ratio^2^	e.e./%^3^
1	AlCl_3_	**8**	4	traces	—	—
2	FeCl_3_	**8**	4	<5	—	—
3	Cu(OTf)_2_^1^	**8**	20	—	—	—
4	Cu(OTf)_2_^1^	**4**	20	<5	—	—
5	Cu(OTf)_2_^1^	**9**	20	31	90/10	<1
6	Cu(OTf)_2_^1^	**5**	20	59	82/18	<1
7	ZnI_2_	**5**	4	23	87/13	<1
8	ZnI_2_^1^	**5**	20	58	87/13	<1
9	Cu(OTf)_2_^1^	**6**	20	21	86/14	12 (*R*)
10	ZnI_2_^1^	**6**	20	30	83/17	24 (*R*)
11	Cu(OTf)_2_^1^	**7**	20	57	82/18	8 (*R*)
12	ZnI_2_^1^	**7**	20	56	88/12	10 (*R*)
13	Cu(OTf)_2_^4^	**7**	20	51	83/17	8 (*R*)

The reactions were performed with 10% of both catalyst and ligand, at 0°C in dichloromethane, except in the mentioned cases. ^1^0°C to room temp.; ^2^measured by ^1^H-NMR; ^3^measured by chiral gaseous chromatography. The absolute configuration was determined by comparison with the [*α*]_*D*_ value of an authentic sample; [15] ^4^10% of catalyst and 20% of ligand.
