# Supplementary material for: New L-Serine Derivative Ligands as Cocatalysts for Diels-Alder Reaction
Source: ISRN Org Chem. 2013 Dec 5;2013:217675. doi: 10.1155/2013/217675 (PMC3870085; doi:10.1155/2013/217675)
Supplement: Supplementary file 1 — This material contains NMR spectra and results of Diels-Alder reactions using BOX ligands. [file 217675.f1.pdf]

New L-serine derivative ligands as co-catalysts for Diels-Alder reaction

Carlos A. D. Sousa,[a] *José E. Rodríguez-Borges*[b] *and Cristina Freire*[a*]

[a] REQUIMTE, Departamento de Química e Bioquímica, Faculdade de Ciências da Universidade do Porto, Rua do Campo Alegre, 4169-007 Porto, Portugal

[b] Centro de Investigação em Química, Departamento de Química e Bioquímica, Faculdade de Ciências da Universidade do Porto Rua do Campo Alegre, 4169-007 Porto, Portugal

Supplementary data

Table of contents

NMR spectra of compounds pag. SI-2

Diels-Alder reactions using BOX ligands as co-catalysts…………………….…... pag. SI-7

NMR spectra

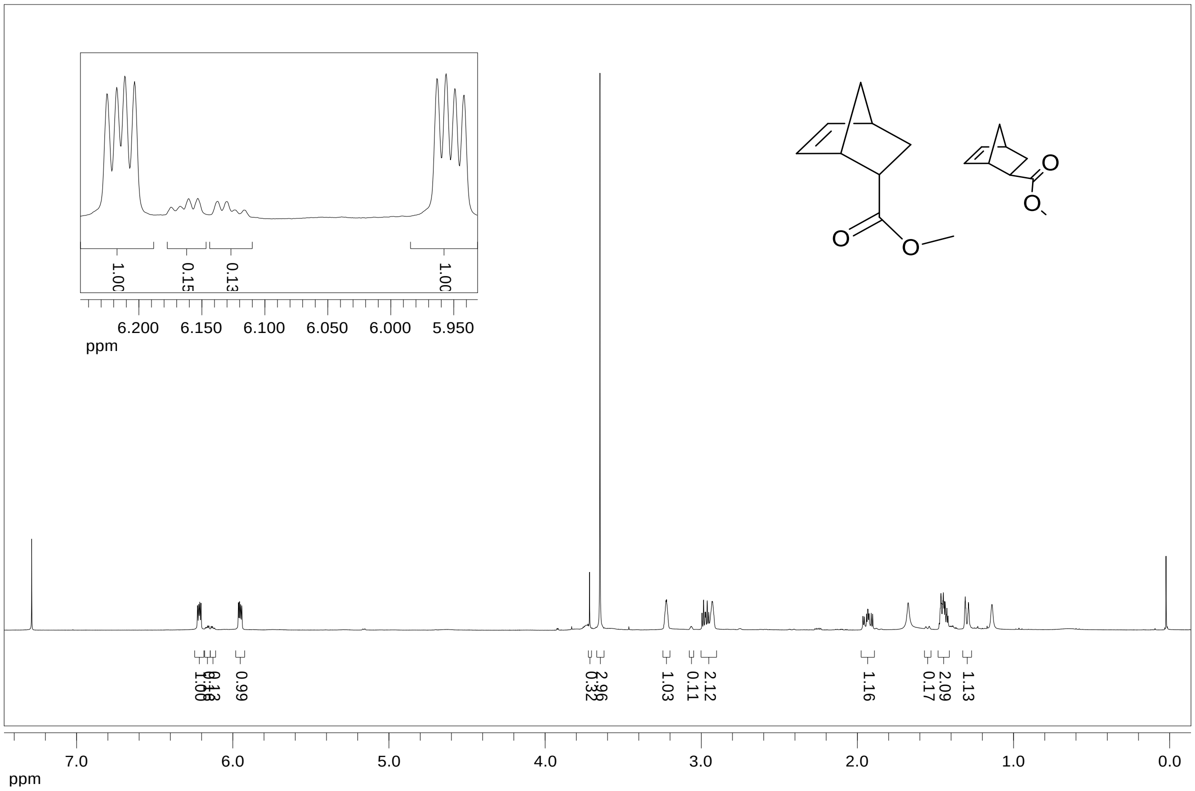

Fig. S1. ^{1}H-NMR spectra of a Diels-Alder reaction mixture catalyzed by ZnI_2, after extraction and evaporation of the volatiles, the **3-*endo*** adduct being majority over the **3-*exo***.

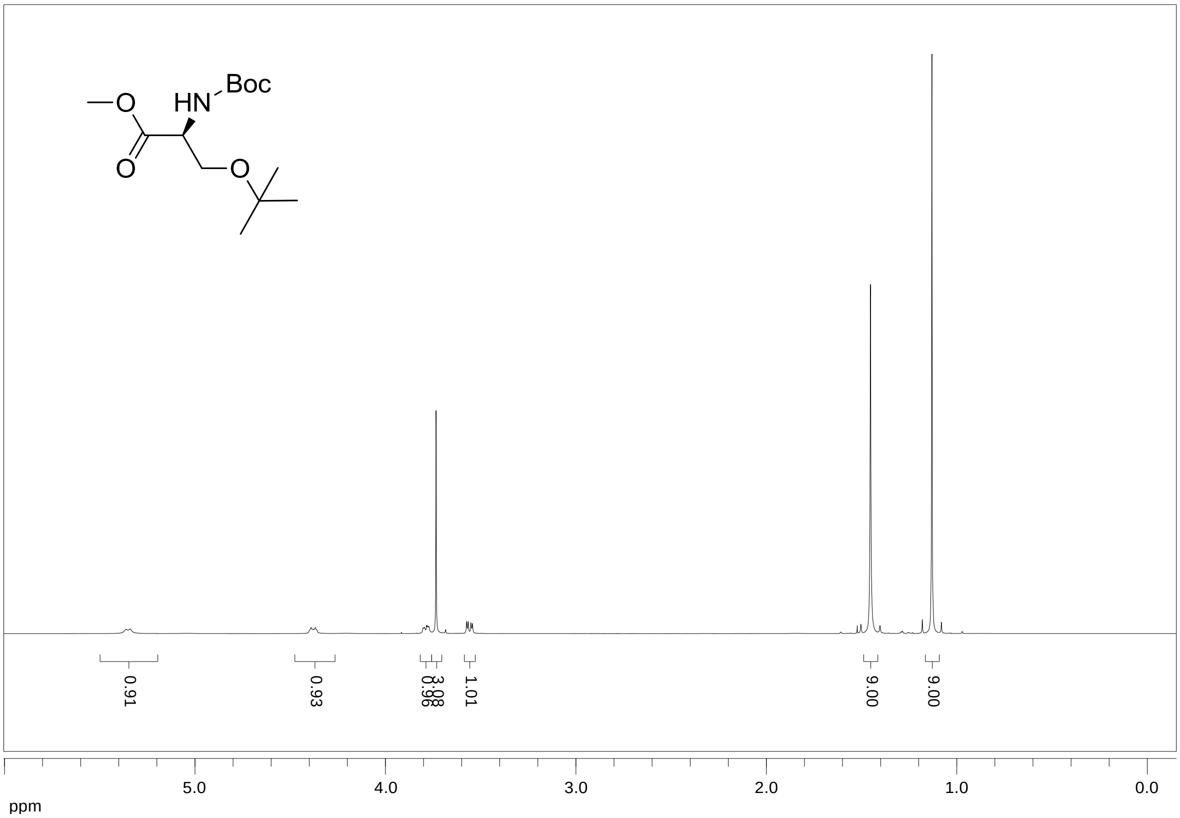

Fig. S2. ^{1}H-NMR spectra of ligand **4**.

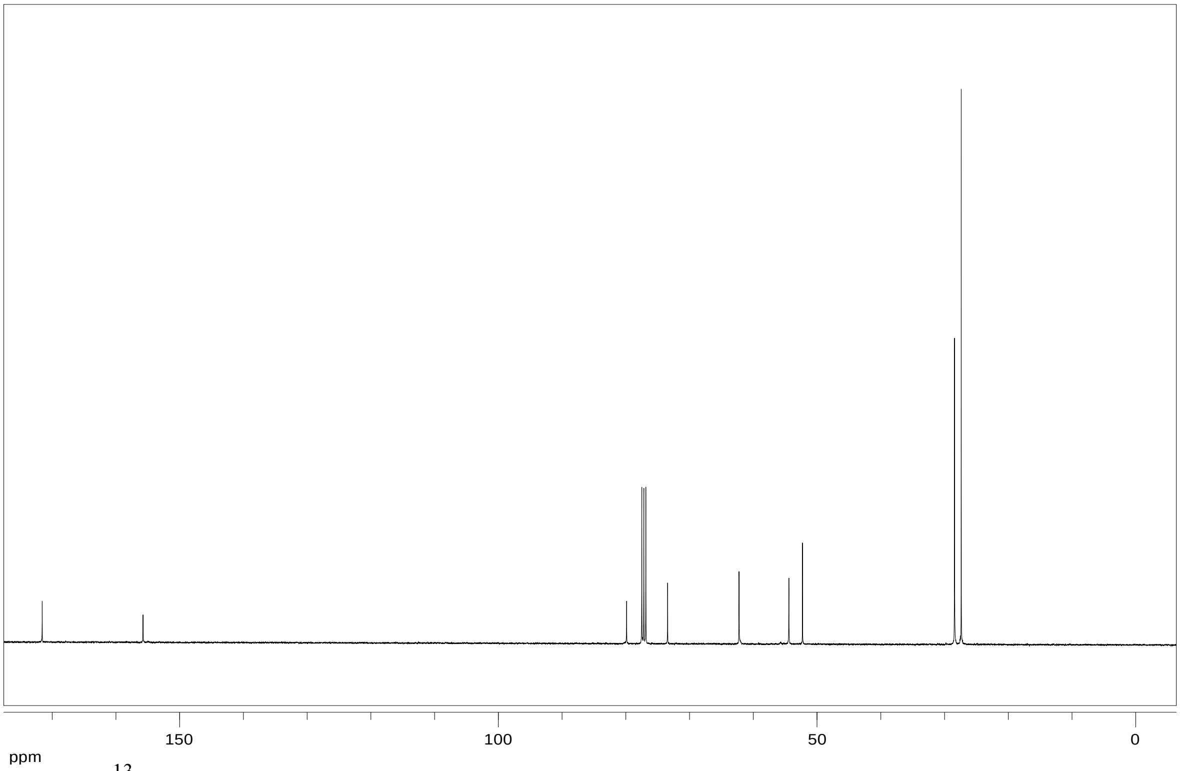

Fig. S3. ^{13}C-NMR spectra of ligand **4**.

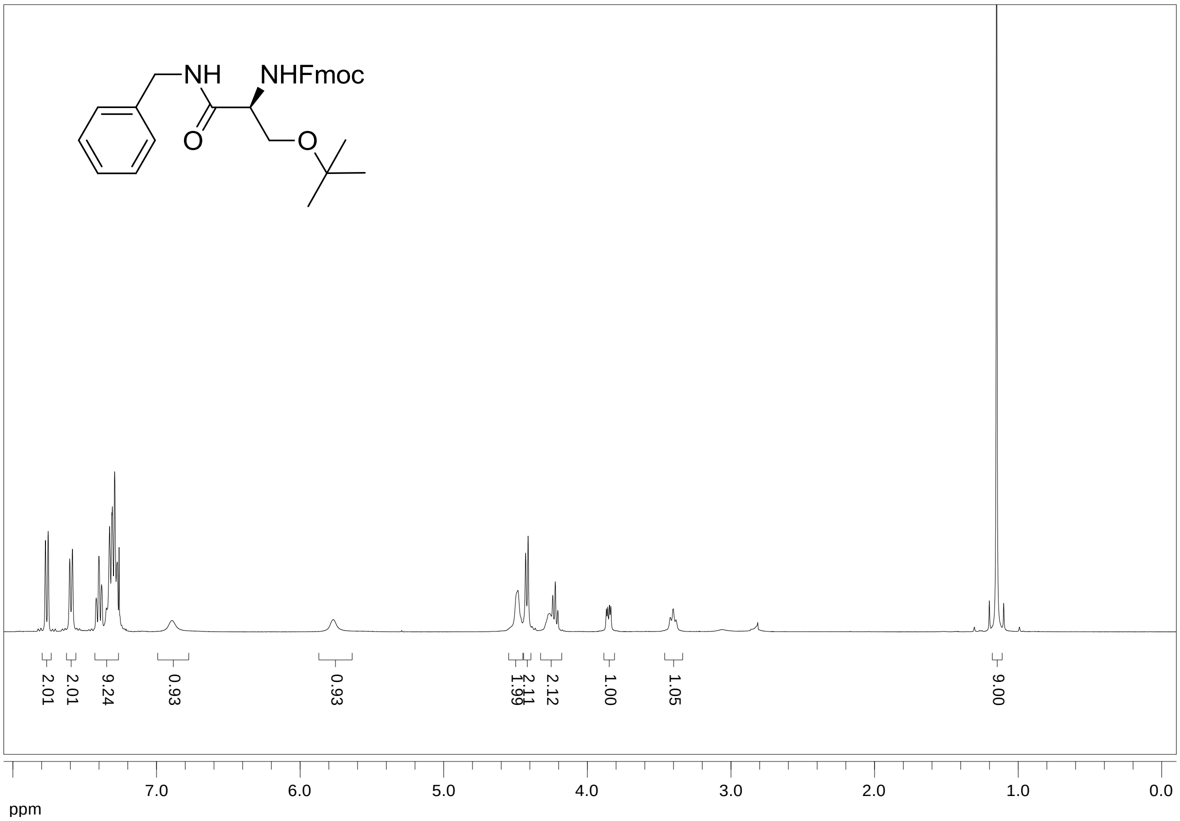

Fig. S4. ^{1}H-NMR spectra of serine derivative ligand **5**.

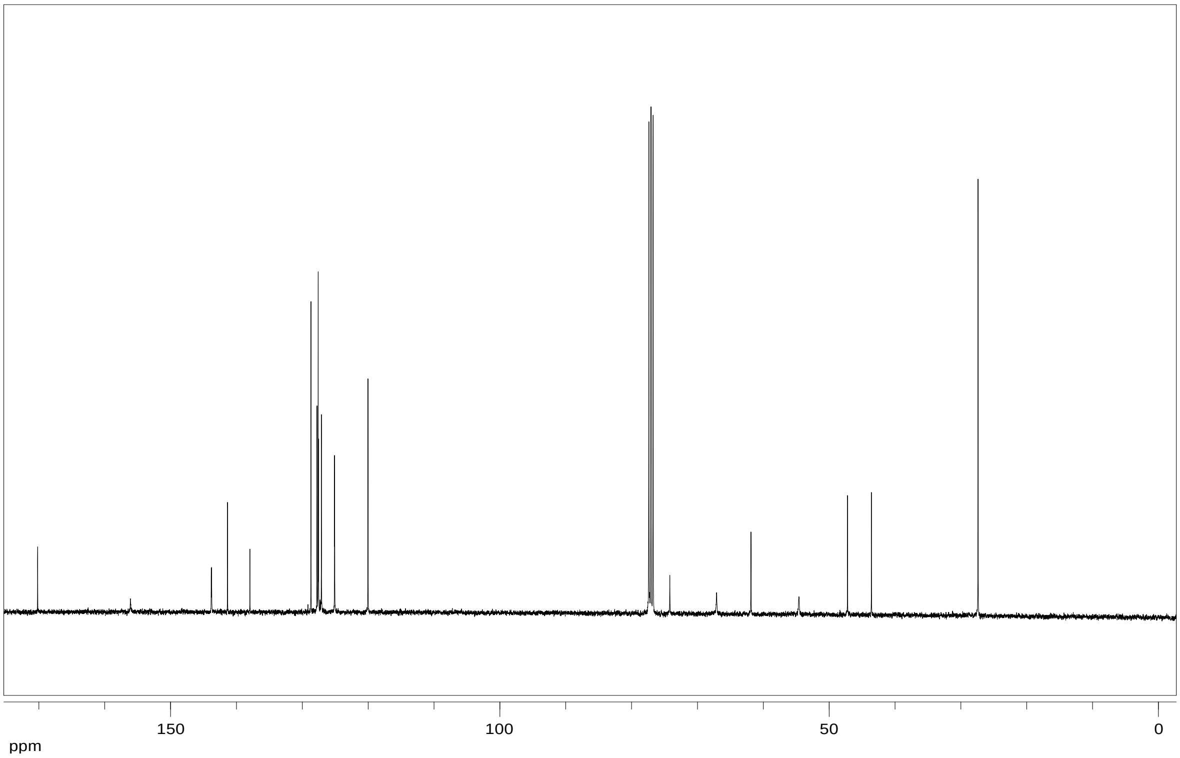

Fig. S5. ^{13}C-NMR spectra of serine derivative ligand **5**.

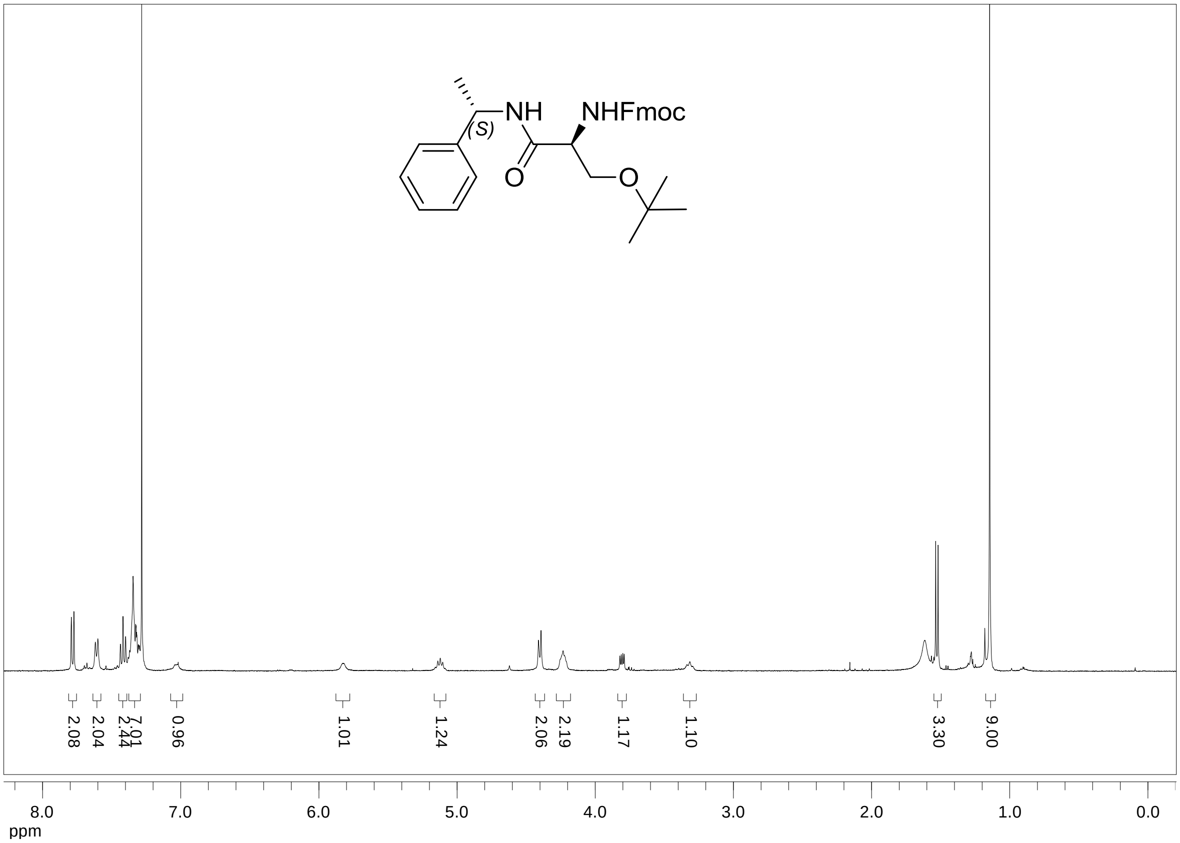

Fig. S6. ^{1}H-NMR spectra of serine derivative ligand **6**.

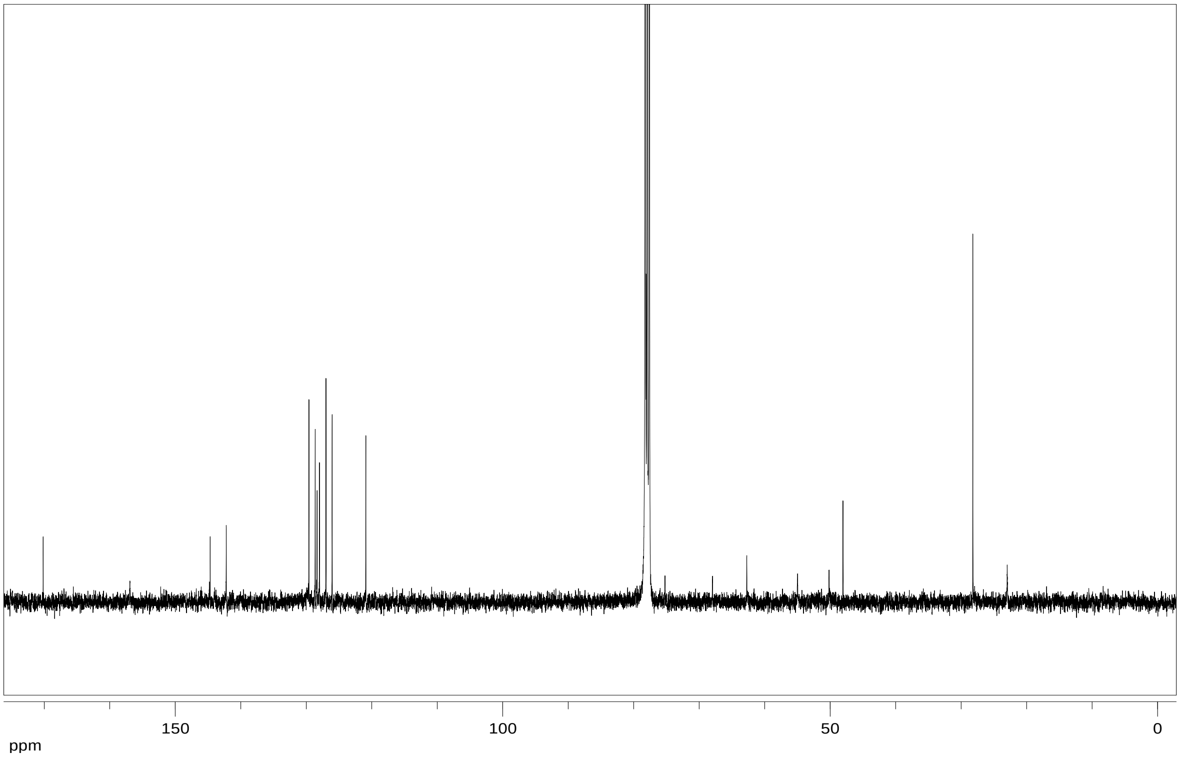

Fig. S7. ^{13}C-NMR spectra of serine derivative ligand **6**.

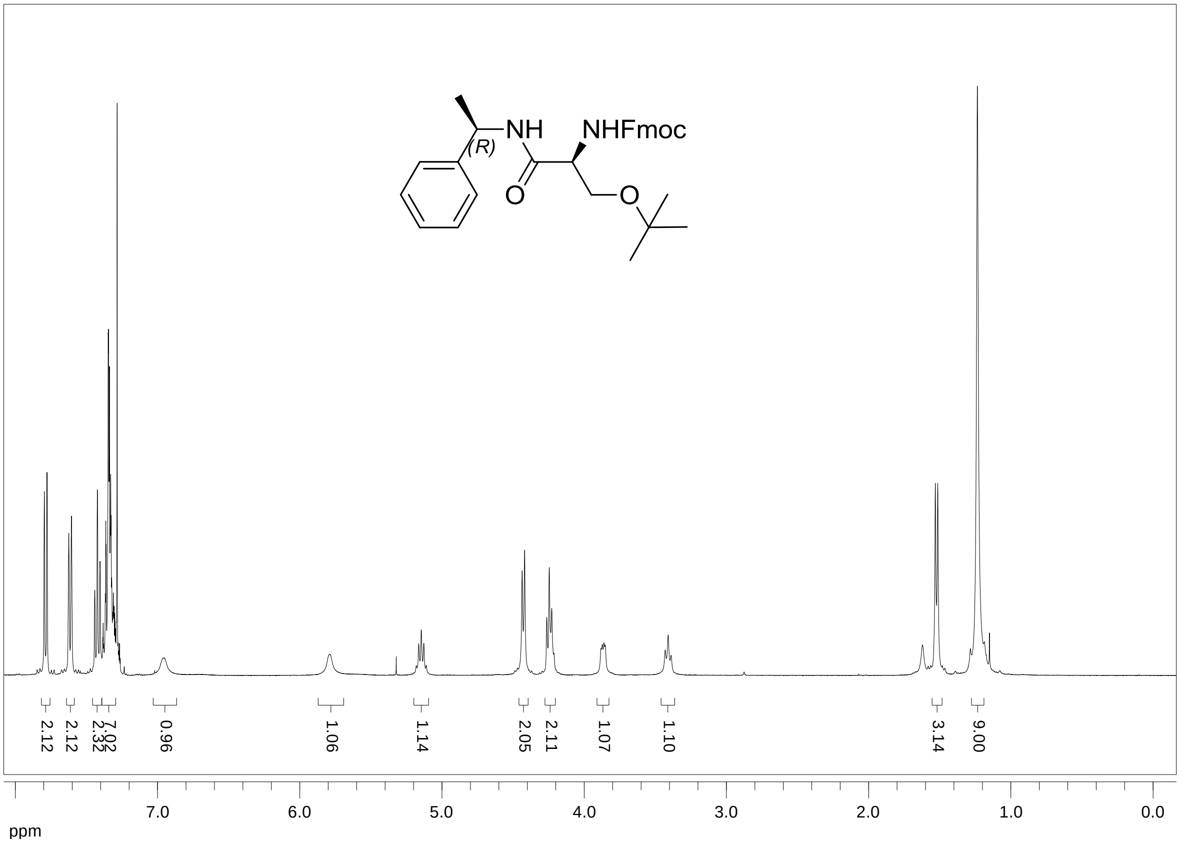

Fig. S8. ^{1}H-NMR spectra of serine derivative ligand **7**.

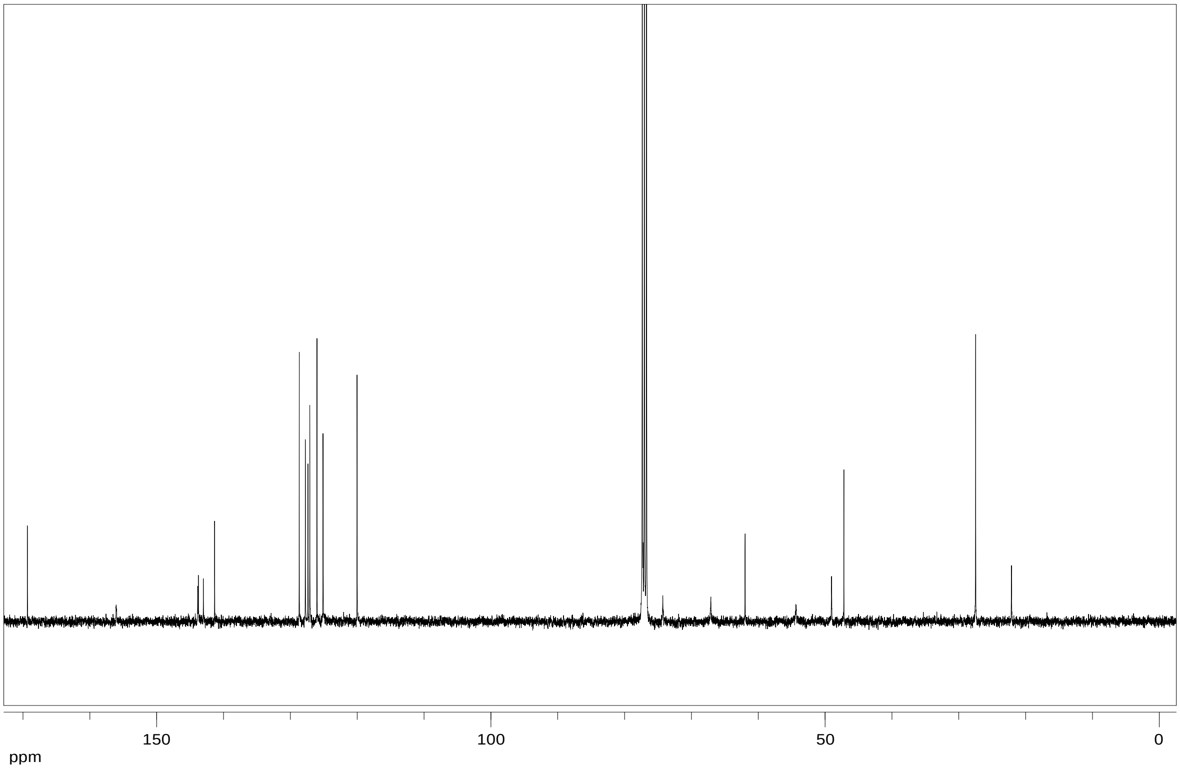

Fig. S9. ^{13}C-NMR spectra of serine derivative ligand **7**.

Diels-Alder reactions using BOX ligands as co-catalysts

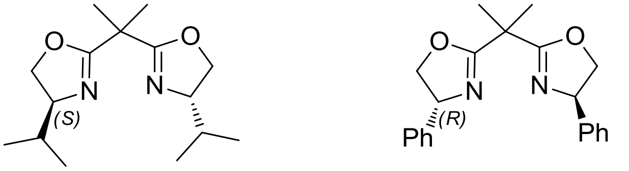

Fig. S10. Structure of BOX ligand **4**, 2,2-bis((4*S*)-4-isopropyloxazoline)propane and **5**, 2,2-bis((4*R*)-4-phenyloxazoline)propane, tested as co-catalysts in Diels-Alder reaction between CPD and methyl acrylate.

Table 2. Results of the the Diels-alder reaction between CPD and methyl acrylate, yield, *endo*/*exo* ratio of adduct **3** and enantiomeric excess (e.e.) using BOX ligands **11** and **12**.

**entry**	**catalyst**	**ligand**	η / %	***endo*/*exo* ratio** [a]	**e.e. / %** [b] (configuration)
1	$Cu(OTf)_2$	**11**	16	83/17	87 (*R*)
2	ZnI_2	**11**	10	86/14	34 (*R*)
3	$Cu(OTf)_2$	**12**	58	83/17	18 (*R*)
4	ZnI_2	**12**	13	83/17	28 (*R*)
5	$Cu(OTf)_2$ [c]	**12**	23	84/16	12 (*R*)

The reactions were performed with 10 % of both catalyst and ligand during 20 h, the temperature allowed to rise from 0 ºC to room temperature, following the procedure described before. *a*- measured by ^{1}H-NMR; *b*- measured by chiral GC; *c*- 15% of ligand and 10% of catalyst, during 20 h, were used.
